# 3’UTR polymorphisms in *NRAMP1* are associated with the susceptibility to pulmonary tuberculosis: A MOOSE-compliant meta-analysis

**DOI:** 10.1097/MD.0000000000015955

**Published:** 2019-06-07

**Authors:** Yang Liu, Erjiang Zhao, Lin Zhu, Danning Zhang, Zhe Wang

**Affiliations:** a Henan Provincial Center for Disease Prevention and Control; b Affiliated Cancer Hospital of Zhengzhou University; c Henan Red Cross Blood Center, Zhengzhou, China.

**Keywords:** meta-analysis, NRAMP1, polymorphism, pulmonary tuberculosis

## Abstract

Many studies have investigated the association between the 3’UTR polymorphism in natural resistance-associated macrophage protein 1 (*NRAMP1*) and the risk of pulmonary tuberculosis (PTB), Revealing inconclusive results. This study aimed to investigate the correlation between the *NRAMP1* 3’UTR polymorphism and the risk of PTB.

This meta-analysis included 29 case–control studies to better and comprehensively assess this correlation. Pooled odds ratios (ORs) and 95% confidence interval (95% CIs) were calculated to assess the strength of the association.

These 29 case–control studies included 4672 cases and 6177 controls. The *NRAMP1* 3’UTR polymorphism displayed a significant positive correlation with the risk of PTB in 3 models (for del/del vs ins/ins: OR = 1.22, 95% CI = 1.01–1.47; for Ins/del vs ins/ins: OR = 1.19, 95% CI 1.08–1.30; for Ins/del + del/del vs ins/ins: OR = 1.25, 95% CI = 1.08–1.45). A stratified analysis by ethnicity revealed that the *NRAMP1* 3’UTR polymorphism was associated with an increased risk of PTB in the Asian population, but not in Caucasian, African, and South American populations.

The present results indicate that the *NRAMP1* 3’UTR polymorphism may be considered a risk factor for PTB in the Asian population.

## 1 Introduction

Tuberculosis (TB), a chronic infectious disease caused by *Mycobacterium tuberculosis*, is a major cause of worldwide mortality and morbidity. In 2016, 10.4 million new cases were reported, and 1.7 million individuals died of TB. Most of the individuals with TB reportedly reside in India China, Philippines, Pakistan, Nigeria, and South Africa.^[1]^ The precise mechanisms underlying TB pathogenesis remain unknown. Weakened immunity, human immunodeficiency virus (HIV) infections, alcohol abuse, advanced age, chronic corticosteroid therapy, diabetes, socio-economic status, and malnutrition are the prominent risk factors of TB.^[2]^ Not all individuals exposed to the similar risk factors develop tuberculosis, thus suggesting the involvement of genetic factors, including single-nucleotide polymorphisms, in TB pathogenesis.

When *M tuberculosis* infects the body, the immune system produces a barrage of defensive protein molecules, recruiting phagocytic cells, which eliminate the pathogen. Natural resistance-associated macrophage protein 1 (*NRAMP1)* is also referred to as the solute carrier family 11 proton-coupled divalent metal ion transporter membrane1 (*SLC11A1*), which plays an important role in the immune response to mycobacterial infections. *NRAMP1* encodes a divalent transition metal (Fe and Mn) transporter that localizes on the lysosomal membrane.^[3]^ Iron is an essential mycobacterial nutrient that also influences the generation of reactive oxygen and nitrogen intermediates. Therefore, NRAMP1 is involved in resistance to intracellular pathogens, including Leishmania, Salmonella, and Mycobacteria. *NRAMP1* is associated with various infectious diseases and inflammatory diseases. It primarily contains 4 polymorphisms, rs17235416 (3’UTR), rs17235409 (D543N), rs3731865 (INT4), and rs34448891 (5=(GT)n). Among these polymorphisms, 3’UTR polymorphisms have been widely investigated for their association with TB. A functionally significant TGTG del allele in *NRAMP1* leads to reduced production of NRAMP1 when compared to the TGTG+ allele and may be correlated with the risk of TB.

Numerous studies have investigated the association between the *NRAMP1* 3’UTR polymorphism and the risk of pulmonary tuberculosis (PTB) in different regions,^[4–29]^ revealing inconclusive results. A case–control study by Medapati et al^[29]^ reported that the *NRAMP1* 3’UTR polymorphism is significantly associated with the susceptibility to TB among Indian individuals. However, Jafari^[28]^ reported no significant association between these 2 aspects. Hence, the present meta-analysis aimed to better and comprehensively assess the correlation between the *NRAMP1* 3’UTR polymorphism and the risk of PTB.

## 2 Materials and methods

### 2.1 Search strategy

We performed a comprehensive search in PubMed, Elsevier, and the Cochrane Library (update till January 1, 2019), using the following keywords

(“tuberculosis” or “TB” or “mycobacteria”), (“polymorphism” or “mutation” or “variant”), and (“Natural resistance associated macrophage protein 1” or “NRAMP1” or “Solute carrier family 11A member 1 or SLC11A1” or “rs17235416”). Articles in all languages were included. Furthermore, additional related articles were identified through screening of the references of relevant articles. In case of a duplicate publication, the largest study was selected. The present study was conducted in accordance with the PRISMA guidelines for systematic reviews and meta-analyses.^[30]^ Ethical approval was not necessary since this study is a meta-analysis.

### 2.2 Inclusion and exclusion criteria

Following were the inclusion criteria of this meta-analysis:

(1) case–control or cohort studies on the *NRAMP1* 3’UTR polymorphism and the risk of PTB;
(2) sufficient data estimating odds ratios (ORs) with 95% confidence intervals (95% CIs).

The major exclusion criteria were as follows:

(1) meta-analyses, letters, reviews, or editorial comments;
(2) studies were not irrelevant to TGTG ins/del;
(3) not PTB;
(4) studies with insufficient data;
(5) other than case–control study;
(6) genotype distributions were not in accordance with Hardy–Weinberg equilibrium (HWE) in the control group.

### 2.3 Data extraction and quality assessment

For all studies, EZ and LZ extracted the data independently and reached a consensus. Any disagreement was resolved through discussion among the 3 authors. The following information was collected from all eligible studies: the first author, year of publication, country of origin, ethnicity, source of controls, number of case and control subjects, HIV status, genotype frequencies in case and control groups, and HWE. We evaluated the quality of all of the studies included according to Newcastle–Ottawa scale (NOS).^[31]^ The NOS contains 3 categories which are selection(0–4 points), comparability(0–2 points), and exposure (0–3 points). The total scores ranged from 0 to 9.

### 2.4 Statistical analysis

The association between the *NRAMP1* 3’UTR polymorphism and the risk of PTB was evaluated using crude ORs with 95% CIs. To assess heterogeneity, the Cochrane Q test and the I-squared (I^2^) metric were used. If the I^2^ metric was <50%, indicating a lack of heterogeneity among studies, A fixed-effects model (the Mantel–Haenszel method) was used. Otherwise, the random-effects model (the DerSimonian and Laird method) was applied.^[32]^ In addition, subgroup analyses based on ethnicity (Asian, Caucasian, African, and South American) were carried out. Funnel plot analysis and Egger test were conducted to identify a publication bias of the meta-analysis.^[33]^ All statistical analyses were performed using STATA 11.0 software (STATA Corp., College Station, TX) and *P* values were 2-tailed.

## 3 Results

### 3.1 Characteristics of eligible studies

**Figure 1:**
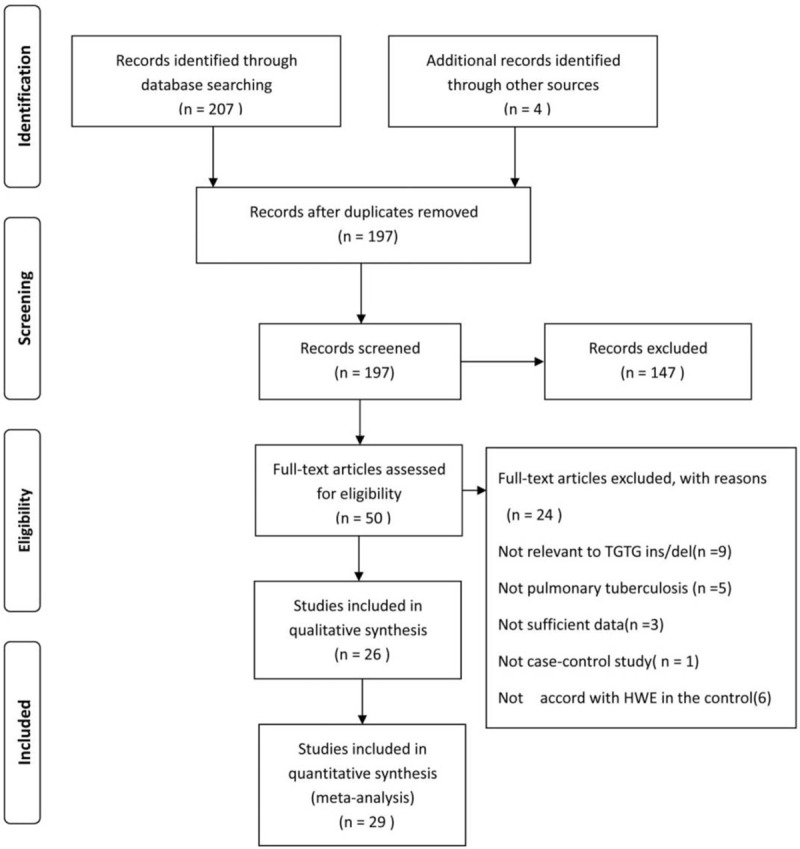
Flow chart of included studies in the current meta-analysis.

In accordance with the flowchart shown in Figure 1, 211 studies were retrieved through our initial literature search. After excluding duplicate articles, reviews, letters, comments, and irrelevant studies, 50 studies remained for further evaluation. After reading full-text articles, 24 studies were excluded. Finally, 26 eligible studies including 29 case–control studies, including 4672 cases and 6177 controls were included (Fig. 1). The major characteristics of identified studies were summarized in Table 1. These studies included 18 studies on Asians, 4 studies on Caucasians, 5 studies on Africans, and 2 studies on South American populations.

**Table 1:**
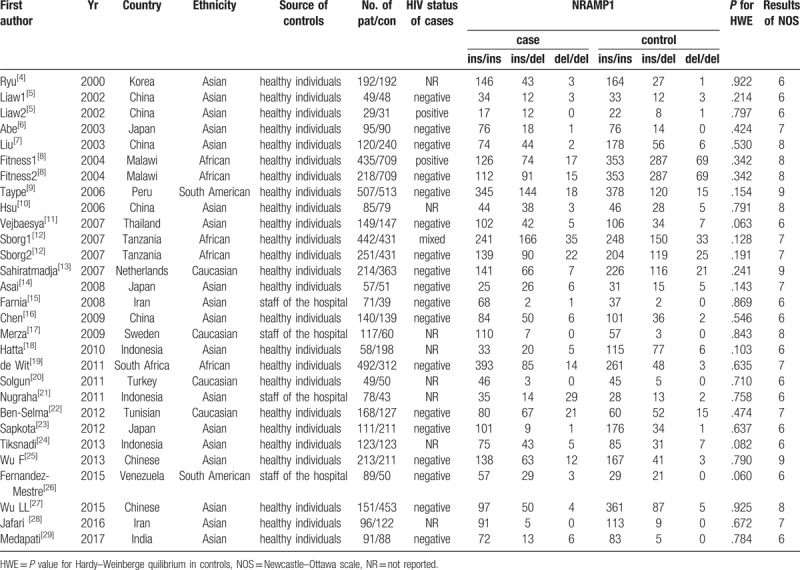
Main characteristics of studies included in this meta-analysis.

### 3.2 Quantitative synthesis

**Figure 2:**
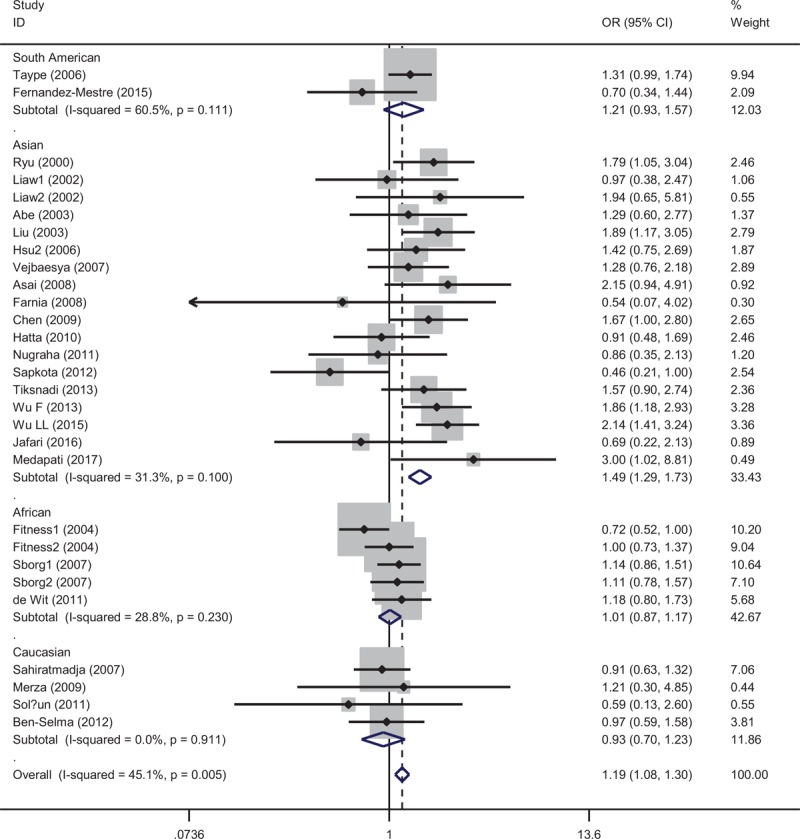
Forest plot for the association between 3’UTR polymorphism and pulmonary tuberculosis risk for the Ins/del genotype compared with the ins/ins genotype In a stratified analysis by ethnicity. CI = confidence interval, OR = odds ratio.

Table 2 summarizes the results of this meta-analysis. Overall, the *NRAMP1* 3’UTR polymorphism was significantly associated with the risk of PTB in 3 models (for del/del vs ins/ins: OR = 1.22, 95% CI = 1.01–1.47; for Ins/del vs ins/ins: OR = 1.19, 95% CI 1.08–1.30; for Ins/del + del/del vs ins/ins: OR = 1.25, 95% CI = 1.08–1.45); however, the recessive model did not reveal this significant association (for del/del vs ins/ins + Ins/del: OR = 1.18, 95% CI 0.98–1.42). In a stratified analysis by ethnicity, the present meta-analysis revealed that the *NRAMP1* 3’UTR polymorphism was associated with an increased risk of PTB in the Asian population (for del/del vs ins/ins: OR = 2.08, 95% CI = 1.45–2.98; for Ins/del vs ins/ins: OR = 1.49, 95% CI 1.29–1.73, Figure 2; for Ins/del + del/del vs ins/ins: OR = 1.57, 95% CI = 1.36–1.18; for del/del vs ins/ins + Ins/del: OR = 1.89, 95% CI 1.33–2.69), but not in the Caucasian, African, and South American populations. In a stratified analysis by HIV status, the *NRAMP1* 3’UTR polymorphism was significantly associated with the risk of PTB in HIV- individuals in 2 models (for Ins/del vs ins/ins: OR = 1.26, 95% CI = 1.06–1.49; for Ins/del + del/del vs ins/ins: OR = 1.28, 95% CI = 1.07–1.54), but not in the HIV+ individuals.

**Table 2:**
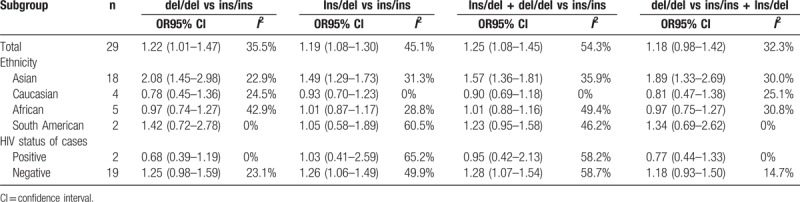
Summary ORs and 95% CI of Association between 3’UTR polymorphism and pulmonary tuberculosis risk.

### 3.3 Publication bias

**Figure 3:**
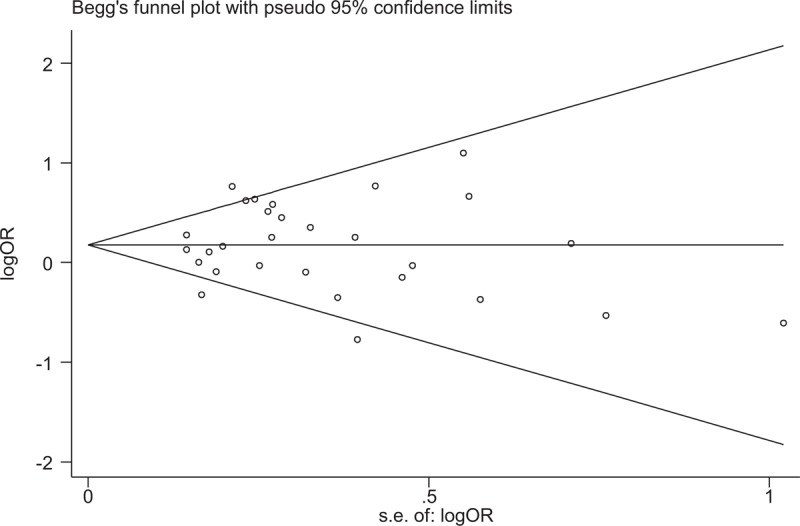
Funnel plot of the association 3’UTR polymorphism and pulmonary tuberculosis risk for the del/del genotype compared with the ins/ins genotype in overall populations. OR = odds ratio, se = standard error.

Funnel plot analysis and Egger's test were conducted to assess publication bias. Egger test did not reveal any evidence of publication bias in the Ins/del versus ins/ins models (*t* = 0.14, *P* = .888) and in the dominant model (*t* = 0.81, *P* = .423); however, publication bias was detected in the del/del vs. ins/ins models (*t* = 2.65, *P* = .014, Fig. 3) and in the recessive model (*t* = 2.43, *P* = .023).

## 4 Discussion

The innate immune response activates early events during a *M tuberculosis* infection. *NRAMP1* plays an important role in the immune response to a mycobacterial infection. Therefore, this meta-analysis aimed to better and comprehensively assess the correlation between the *NRAMP1* 3’UTR polymorphism and the risk of PTB. The present study reports a significant correlation between the *NRAMP1* 3’UTR polymorphism and the risk of PTB in overall and Asian populations.

Many epidemiological studies have investigated the association between the *NRAMP1* 3’UTR polymorphism and the risk of PTB. In 2006, a meta-analysis by Li et al^[34]^ reported that 3’UTR polymorphisms are significantly associated with the risk of PTB. Subsequently, a meta-analysis by Meilang et al^[35]^ on PTB and extra-PTB reported that 3’UTR polymorphisms increased the risk of TB in comparison with their corresponding common alleles. Furthermore, stratified analyses by ethnicity, which assessed the forms of TB, confirmed the results of Li et al. This meta-analysis includes 29 recent case–control studies including 4672 cases and 6177 controls to better and comprehensively assess the correlation between the *NRAMP1* 3’UTR polymorphism and the risk of PTB. The present results are similar to those of 2 previous meta-analyses. The 3’UTR del/del and ins/del genotypes were significantly associated with a higher risk than the 3’UTR ins/ins genotype.

In 2000, the association between the *NRAMP1* 3’UTR polymorphism and the risk of PTB was first investigated by Ryu et al.^[5]^ They revealed a significant relationship of the ins/del genotype variant with the risk of PTB among Koreans, but not in the del/del genotype. A case–control study by Medapati et al^[29]^ reported that the *NRAMP1* 3’UTR polymorphism is significantly associated with the susceptibility to TB in the Indian population. However, Taype et al^[9]^ reported did not report an association in the Peruvian population. These contradictory results may be explained on the basis of the present results, suggesting that the risk of PTB conferred by the variant allele may be modified by race. The present meta-analysis reported that the *NRAMP1* 3’UTR polymorphism was associated with an increased risk of PTB in the Asian population, but not in Caucasian, African, and South American populations.

Despite the strengths of the present study, some limitations should be acknowledged. First, upon subgroup analysis by ethnicity, the *NRAMP1* 3’UTR polymorphism was not associated with the risk of PTB in Caucasian, African, and South American populations. However, the sample size was relatively small in Caucasian, South American, and African populations, thus potentially decreasing the statistical power of the results to establish an actual association. Second, publication bias was detected in the del/del versus ins/ins models and the recessive model in overall analysis; hence, the present results should be considered with caution. Third, PTB is a complex disease; however, we did not carry out subgroup analysis to analyze potential gene-gene and gene-environment interactions because of insufficient data. Fourth, this meta-analysis was performed with a candidate gene strategy. Genome-wide association studies (GWAS) scanning entire genomes for genetic variation include immense amounts of SNPs and have been designed for the same ethnic background and study design. A GWAS by Zheng et al^[36]^ reported that 2 loci 14q24.3 (rs12437118, Pcombined = 1.72 × 10−11, OR = 1.277, ESRRB) and 20p13(rs6114027, Pcombined = 2.37 × 10.11, OR = 1.339, TGM6) were significantly associated with TB in Han Chinese individuals. Miao et al^[37]^ reported that rs9272461 is significantly associated with the risk of PTB in various genetic models. Hence, GWAS are required to further validate the present results.

In conclusion, the present results indicate that the *NRAMP1* 3’UTR polymorphism may be associated with an increased risk of PTB in the Asian population, but not in the Caucasian, African, and South American populations. Well-designed epidemiological studies with larger sample sizes are needed to verify the present findings.

## Acknowledgments

We would like to thank Editage [www.editage.cn] for English language editing.

## Author contributions

**Data extraction:** Erjiang Zhao, Lin Zhu

**Formal analysis:** Erjiang Zhao, Danning Zhang

**Investigation:** Yang Liu, Lin Zhu, Dan Ning Zhang.

**Methodology:** Yang Liu, Erjiang Zhao, Dan Ning Zhang, Zhe Wang, Danning Ding.

**Resources:** Yang Liu.

**Software:** Erjiang Zhao, Lin Zhu.

**Writing – original draft:** Yang Liu, Erjiang Zhao.

**Writing – review & editing:** Yang Liu.

Yang Liu orcid: 0000-0002-3877-9203.
